# High‐Frequency Ultrasonography in Cutaneous Lupus Erythematosus

**DOI:** 10.1111/srt.70208

**Published:** 2025-08-11

**Authors:** Aneta Karasińska, Aleksandra Dańczak‐Pazdrowska, Adriana Polańska

**Affiliations:** ^1^ Department of Dermatology Poznan University of Medical Sciences Poznan Poland

**Keywords:** cutaneous lupus erythematosus, high‐frequency ultrasonography, skin USG, SLEB, subepidermal low echogenic band

## Abstract

**Introduction:**

Cutaneous lupus erythematosus (CLE) is a chronic inflammatory autoimmune disease with not fully understood pathogenic mechanisms. The lesions in CLE are mainly located on the facial skin, that is why searching for noninvasive methods to facilitate differential diagnosis is justified. High‐frequency ultrasonography (HFUS) is a noninvasive diagnostic tool, enabling the assessment of all skin layers without leaving a scar, in contrast to skin biopsy, which is the gold diagnostic standard in this disease. The aim of this study was to assess the usefulness of HFUS in the diagnostic process of patients with CLE, taking into account individual types of the disease, its severity and activity.

**Materials and Methods:**

For the purpose of analyzing the HFUS results, the studied lupus erythematosus (LE) group was divided into three subgroups: acute cutaneous lupus erythematosus (ACLE), subcutaneous lupus erythematosus (SCLE), and chronic cutaneous lupus erythematosus (CCLE). Fifty‐seven active lesions and nine inactive lesions were examined. The analysis was performed using a 20 MHz Dermascan C linear head (version 3) (Cortex Technology; Hadsund, Denmark). In the analysis of the HFUS image, skin thickness, echogenicity of the skin, and the presence and thickness of subepidermal low echogenic band (SLEB) were taken into consideration. The image was compared with the healthy skin of the contralateral area and, if this was not possible, with the skin in the area of the lesion.

**Results:**

HFUS images of 17 patients with active CLE (29.8%) showed the presence of SLEB and a statistically significant relationship between SLEB thickness and disease activity expressed by Cutaneous Lupus Erythematosus Disease Area and Severity Index (CLASI‐A) (*p* = 0.022). The average skin thickness of active foci was significantly greater than the average thickness of healthy skin (*p* = 0.001). Additionally, in the group of patients with CCLE, 55.6% showed increased skin echogenicity, showing its statistically significant inverse correlation with CLASI‐A (*p* = 0.004).

**Conclusions:**

SLEB can be treated as an indicator of the activity of the disease process. HFUS allows the assessment of certain features of healthy and diseased skin without performing a biopsy, but it cannot replace histological examination in the case of CLE diagnosis.

## 1 Introduction

Cutaneous lupus erythematosus (CLE) is a chronic inflammatory autoimmune disease of not fully understood pathogenic mechanisms. The potential risk of progression to systemic lupus erythematosus (SLE) applies to every patient, positively correlating with the duration of the disease [1, 2, 3]. According to current diagnostic criteria, the gold standard in the diagnosis of CLE is histological examination, which often results in the formation of unsightly scar. The lesions in CLE are mainly located on the facial skin—a place that is not very acceptable for invasive methods [1, 3, 4].

High‐frequency ultrasonography (HFUS) is a noninvasive, low‐cost, repeatable diagnostic tool, without contraindications and side effects. It is used in the assessment of both healthy and diseased skin [5, 6, 7, 8]. In the HFUS image of healthy skin, we can distinguish three basic layers: the epidermis echo, the dermis, and the subcutaneous tissue, which corresponds to its anatomical structure. It is also possible to measure their thickness. The assessment of affected skin should be made in comparison with healthy skin, surrounding or contralateral area. The role of HFUS is emphasized not only by dermatologists and radiologists, but also by rheumatologists, as evidenced in publications on its implementation in systemic sclerosis (SSc) [7, 8].

In recent years, the possibilities of using HFUS in dermatology have been expanding [9, 10, 11, 12, 13]. In HFUS image, in inflammatory skin diseases such as atopic dermatitis and psoriasis, we can detect subepidermal low echogenic band (SLEB) indicating the presence of an inflammatory process in the skin. This band thins or disappears as the dermatological condition improves [14]. Moreover, there are reports in the literature regarding the correlation of deviations in HFUS images with histological examination [15, 16]. There are no scientific reports in the literature related to the use of HFUS in CLE.

This study was conducted to assess the usefulness of HFUS in the diagnostic process of patients with CLE, taking into account individual types of the disease, its severity and activity.

## 2 Materials and Methods

The study lupus erythematosus (LE) group consisted of 70 adults, 56 women (80%) and 14 men (20%). The mean age was 52 ± 15.2 years (25–85): 52.6 ± 14.7 for women (25–83) and 49.7 ± 17.6 for men (28–85). The mean disease duration was 9.3 ± 8.3 years (1–37). The inclusion criteria were met in the case of patients with isolated CLE (who did not meet the SLE criteria) and patients with CLE who met the criteria for the diagnosis of SLE [17]. Patients with SLE without any skin symptoms were excluded from the study. Also, nonspecific variants of LE have not been studied. Sixteen patients meeting both Systemic Lupus International Collaborating Clinics (SLICC) and EULAR/ACR criteria were qualified to SLE group [17, 18]. The mean age of patients was 47.4 ± 15.3 years (25–82) and the mean disease duration was 9.6 ± 8.3 years (1–29). On the Systemic Lupus Erythematosus Disease Activity Index (SLEDAI) scale, disease activity ranged from 2 to 15, with an average of 9.56 ± 3.32 points [19]. Acute cutaneous lupus erythematosus (ACLE) was diagnosed in 2 patients (12.5%), discoid lupus erythematosus (DLE) in 10 (62.5%), and subcutaneous lupus erythematosus (SCLE) in 4 patients (25%). There was no coexistence of different CLE forms in any of the patients. The mean value of Cutaneous Lupus Erythematosus Disease Area and Severity Index Activity (CLASI‐A) was 7.88 points (0–26), and Cutaneous Lupus Erythematosus Disease Area and Severity Index Damage (CLASI‐D) 4.50 points (0–13) [20]. Fifty‐four patients were qualified for the isolated form of CLE. In this group, two subgroups—chronic cutaneous lupus erythematosus (CCLE) and SCLE—were distinguished. The mean age of patients was 53.4 ± 15.0 years (25–85) and the mean disease duration 9.3 ± 8.3 years (1–37). The mean value of CLASI‐A was 5.70 points (0–27) and CLASI‐D 4.59 points (0–23).

For the purpose of analyzing the HFUS results, the studied LE group was divided into three subgroups: ACLE, SCLE, and CCLE.

Each patient signed a written consent to the study. The research was approved by the local Bioethics Committee (UMP resolution No. 1231/16, 1243/17).

**FIGURE 1:**
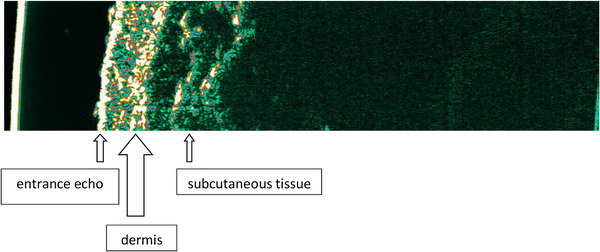
The image of healthy skin in high‐frequency ultrasonography.

HFUS examination was performed using a 20 MHz Dermascan C linear head (version 3) (Cortex Technology; Hadsund, Denmark). After placing the patient in a comfortable, stationary position, gel intended for ultrasound was applied to the examined skin lesion. The head was placed on the skin so that in the obtained image the surface of the imaged skin was located perpendicular to the upper edge of the image. The examination was performed in a type B presentation, with subsequent image recording. The probe scanned the image at 6–8 frames per second, in real time. The speed of the ultrasonic wave in the skin was 1580 m/s. The penetration of the ultrasound beam into the tissue was approximately 10 mm, the axial resolution was 80 µm, and the lateral resolution was 200 µm. Fifty‐seven active lesions and nine inactive lesions were examined, and if the patient had several lesions, the most representative lesion in the opinion of the researcher was selected for examination. A maximum of two lesions were examined in one patient, provided that active and inactive lesions coexisted. In the analysis of the HFUS image, skin thickness, echogenicity of the skin, and the presence and thickness of SLEB were taken into account. The basis for assessing echogenicity was the visual assessment of the HFUS image. However, the average skin thickness and average SLEB thickness were determined automatically using the device software in accordance with the manufacturer's recommendations. The image was compared with the healthy skin of the contralateral area and, if this was not possible, with the skin in the area of the lesion. The HFUS image of healthy skin is shown in Figure 1.

In the last stage, statistical analysis by R program (R Foundation for Statistical Computing, Wirtschaftsuniversität Wien, Austria) was performed. The results were considered statistically significant at *p* < 0.05. In order to characterize individual groups, descriptive statistics of patients’ age and disease duration were presented: arithmetic mean, standard deviation, as well as minimum and maximum values. A linear regression model was assessed to evaluate the effect of CLASI‐A magnitude on SLEB. The relationship between echogenicity and CLASI was examined using Welch's *t*‐test.

## 3 Results

**FIGURE 2:**
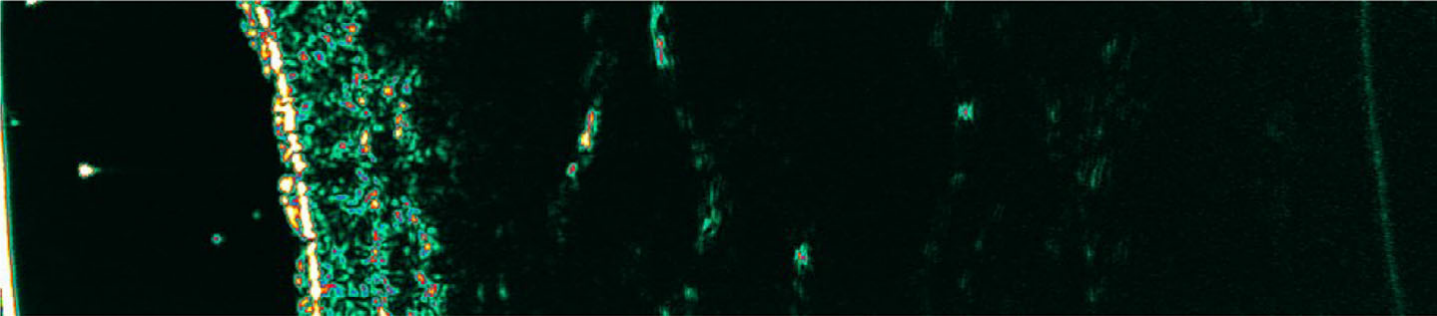
High‐frequency ultrasound image in a patient with acute cutaneous lupus erythematosus (cheek skin). All layers of the skin are visible and the echogenicity of the skin is reduced.

The study was performed in two patients with ACLE in the active phase. Among the abnormalities, decreased skin echogenicity was found in one of them (50%) (Figure 2).

We included 13 patients with SCLE in whom active lesions were observed. The examination was not performed in four patients in the inactive phase. Deviations were detected in nine patients.

**FIGURE 3:**
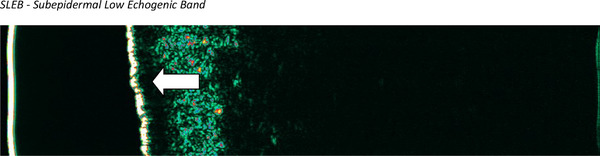
High‐frequency ultrasound image in active subacute cutaneous lupus erythematosus (facial skin). The arrow indicates SLEB. SLEB, subepidermal low echogenic band.

In the active phase, we observed the presence of SLEB in four subjects (30.8%) and decreased skin echogenicity in three subjects (23.1%), group of echogenicity decreased, one patient had additionaly SLEB (7.7%). Increased skin echogenicity was not observed. Nine respondents (69.2%) had skin thickening. The presence of SLEB is shown in Figure 3.

**FIGURE 4:**
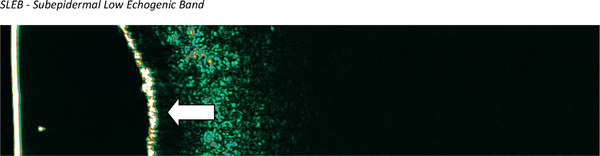
High‐frequency ultrasound image of active discoid lupus erythematosus (facial skin). The arrow indicates SLEB. SLEB, subepidermal low echogenic band.

The study was performed in 51 patients with CCLE, of whom active lesions were observed in 42 and inactive lesions in 9. In CCLE patients in the active phase, we observed the presence of SLEB in 13 subjects (31.0%) and decreased skin echogenicity in 11 subjects (26.2%), group of echogenicity decreased, four patient had additionaly SLEB (9.5%). Increased skin echogenicity was observed in 12 subjects (28.6%). In 19 subjects (21.4%), there were no changes in skin echogenicity. Skin thickening was observed in 25 patients (59.5%). The presence of SLEB is shown in Figure 4.

**FIGURE 5:**
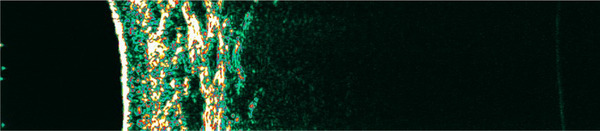
Increased echogenicity of the skin in a patient with discoid lupus erythematosus—inactive focus, with clinically severe scarring (skin of the right preauricular area).

Ultrasound features present in CCLE patients in the inactive phase are following. Five subjects (55.6%) had increased skin echogenicity, including two of them (22.2%) with skin thickening. In four subjects (44.4%) ultrasound images did not differ from those of healthy skin. Increased skin echogenicity was observed, among others, in lesions with clinically visible scarring, as well as in some active lesions (Figure 5).

**FIGURE 6:**
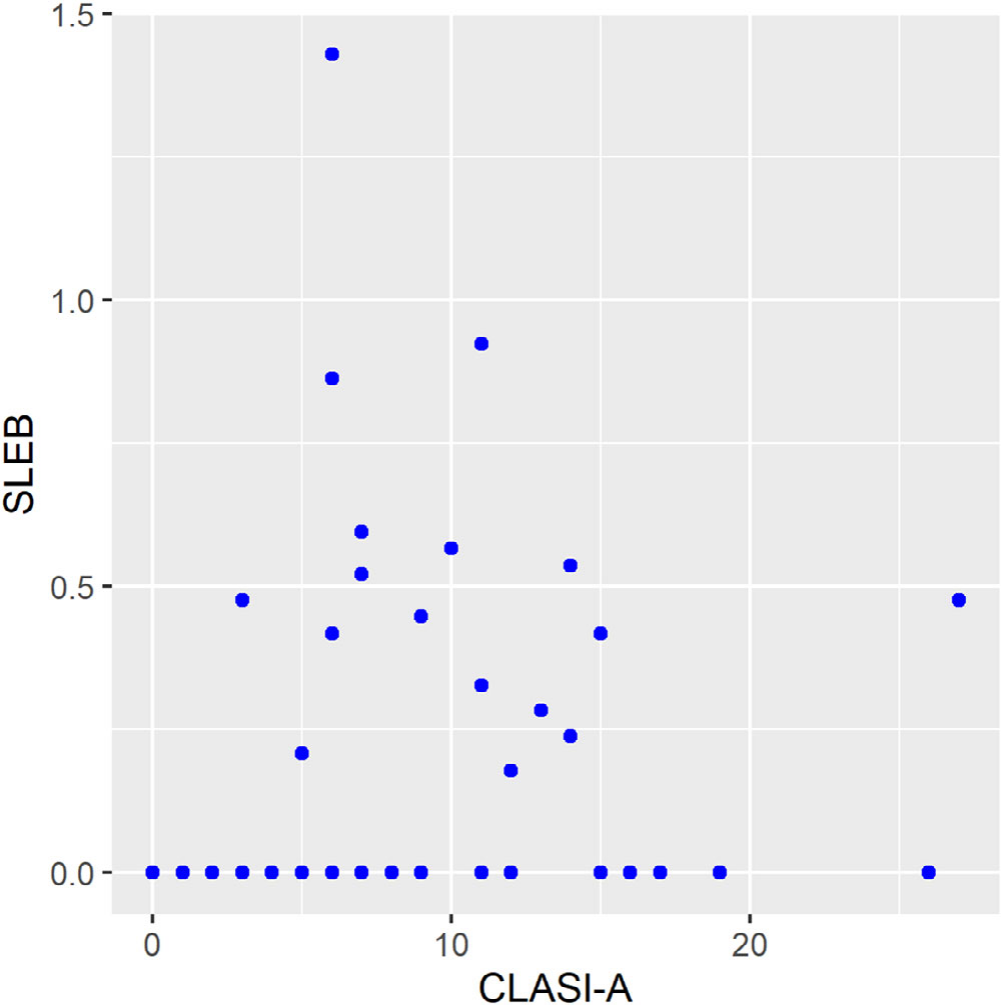
The correlation between Cutaneous Lupus Erythematosus Cutaneous Disease Area and Severity Index (activity) and subepidermal low echogenic band. CLASI‐A, Cutaneous Lupus Erythematosus Cutaneous Disease Area and Severity Index (activity); SLEB, subepidermal low echogenic band.

At a later stage of the study, the relationship between CLASI and SLEB was assessed. The presence of SLEB was detected in 17 patients with active CLE (29.8%), and was not detected in any patient with the inactive form, nor was observed on scans of healthy skin. The thickness of SLEB in active foci ranged from 0 to 1.430 mm, with mean 0.524 mm. A statistically significant relationship was found between CLASI‐A and SLEB (*p* = 0.002). Increasing CLASI‐A by one increases the expected SLEB value by 0.012 mm. The model explains 7.4% of the variation in SLEB. The results are presented in Table 1 and Figure 6.

**TABLE 1:** The correlation between Cutaneous Lupus Erythematosus Disease Area and Severity Index (activity) and subepidermal low echogenic band.

Variable	Evaluation	Standard deviation	*t*‐statistic	*p*
**(Free word)**	0.05	0.045	1.11	0.271
**CLASI‐A**	0.012	0.0053	2.344	0.022
**Model characteristics**	*R* ^2^ = 0.074 *F*[1.68] = 5.492; *p* = 0.022			

Abbreviation: CLASI‐A, Cutaneous Lupus Erythematosus Cutaneous Disease Area and Severity Index (activity).

The assessment of relationship between CLASI‐A and skin thickness revealed no significant correlation for all patients with activity above 0 in any of the studied subgroups.

**FIGURE 7:**
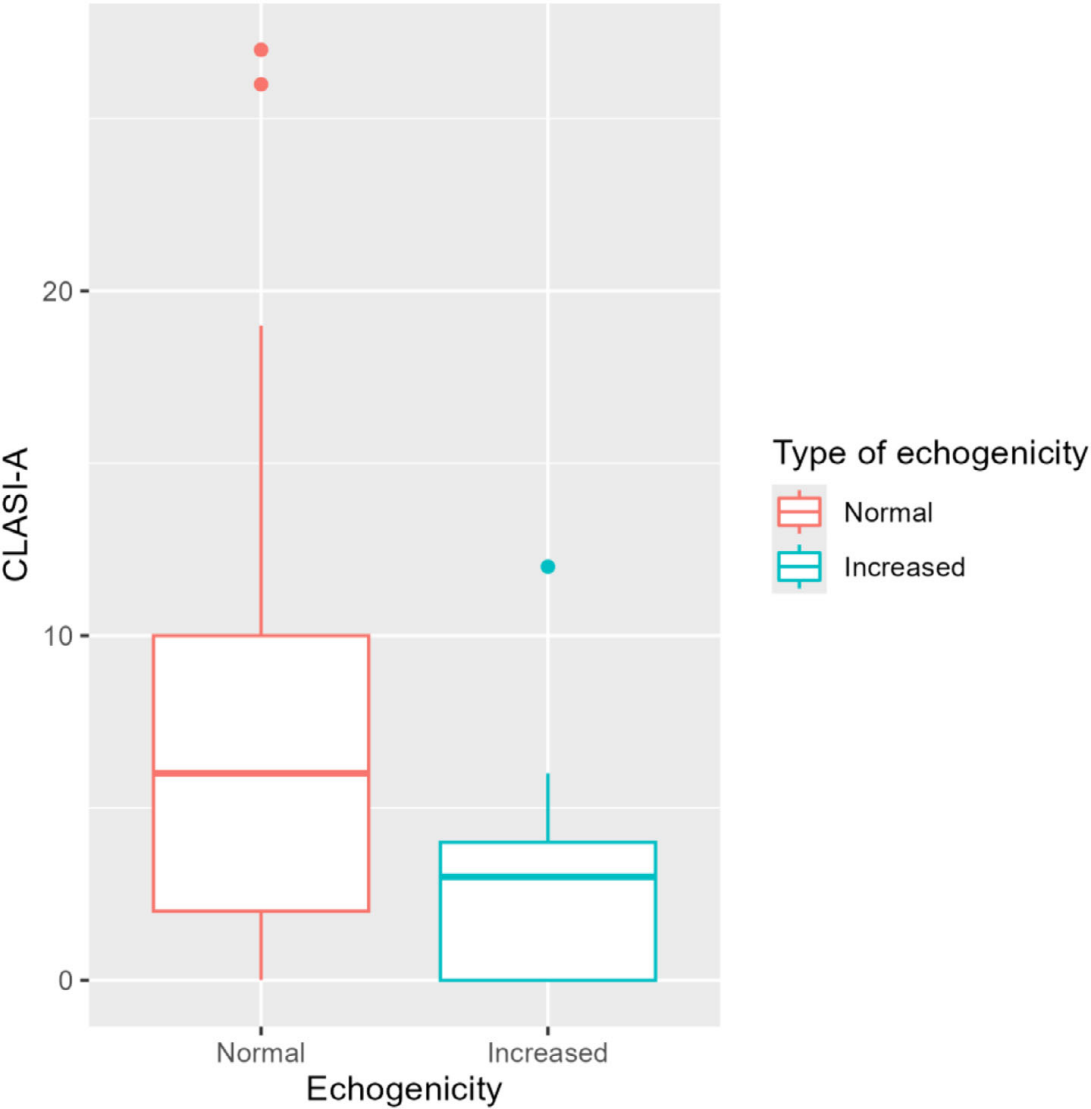
Distribution of the Cutaneous Lupus Erythematosus Disease Area and Severity Index (activity) in groups with normal and increased echogenicity. CLASI‐A, Cutaneous Lupus Erythematosus Disease Area and Severity Index (activity).

There was no significant relationship between decreased echogenicity and CLASI‐A, and between increased and decreased echogenicity and CLASI‐D. However, a statistically significant relationship was found between increased echogenicity and CLASI‐A (*p* = 0.004), with increased echogenicity indicating lower CLASI‐A values (Table 2, Figure 7).

**TABLE 2:** Welch's *t*‐test for normal (group 1) and increased echogenicity groups (group 2).

CLASI‐A			
Average 1	Average 2	*t*	*p*
7.108	3.059	2.989	0.004

Abbreviation: CLASI‐A, Cutaneous Lupus Erythematosus Disease Area and Severity Index (activity).

## 4 Discussion

In the present study, HFUS images were analyzed in patients with various forms of CLE. An attempt was made to assess the correlation of selected parameters with the activity of the disease process. Particular attention was paid to the presence of SLEB, echogenicity, and skin thickness. So far, there is no scientific report on the HFUS image in LE. Our study included two patients with ACLE in the active phase of the disease—in one of them only reduced skin echogenicity was observed. In patients with SCLE in the active phase, four had SLEB (30.8%) and three had reduced skin echogenicity (23.1%). Skin thickening was observed in nine subjects (69.2%). In the CCLE group, 13 patients (31%) had SLEB in the active phase, 11 (26.2%) had reduced skin echogenicity, and 12 (28.6%) had increased skin echogenicity. In four subjects (9.5%), in whom reduced skin echogenicity was observed, SLEB was also observed (9.5%). Skin thickening was observed in 25 patients (59.5%). In the CCLE group in the inactive phase, five patients (55.6%) had increased skin echogenicity, including two of them (22.2%) with skin thickening. However, in the remaining four patients (44.4%), the ultrasound images did not differ from those of healthy skin. Increased skin echogenicity was observed, among others, in lesions with clinically visible scarring, as well as in some active lesions.

SLEB was only demonstrated in patients with active CLE. It was also not observed in healthy skin. SLEB was also found more often in CCLE than in SCLE, but was not observed in ACLE, which may be related to the differences observed in histological examination, where the presence of “interface dermatitis” is least expressed in ACLE, more in SCLE, and most in CCLE [21, 22, 23, 24]. However, this hypothesis requires confirmation in further studies, which will compare the HFUS and histological images. It should be emphasized that there was a statistically significant correlation between SLEB and CLASI‐A (*p* = 0.022)—increasing CLASI‐A by one resulted in an increase in the SLEB value by 0.012 mm. This means that SLEB can serve as an indicator of disease process activity. Of course, SLEB is not a pathognomonic symptom of LE; its presence has been observed in a number of other diseases, including inflammatory and cancerous ones [6, 10, 25, 26].

SLEB is considered an “indicator” of inflammatory or cancer cell infiltration, as well as an expression of swelling of the papillary layer caused by, among others, water accumulation [5, 15, 27]. Its thickness in many cases correlates with the severity of the disease [10, 14, 16]. SLEB is also a characteristic ultrasound feature of skin excessively exposed to ultraviolet rays [5, 28]. An increasing number of studies indicate that SLEB can serve as a parameter assessing the effectiveness of treatment—a reduction in the thickness or disappearance of SLEB in the case of improvement of the local skin condition under the influence of treatment has been demonstrated, among others, in atopic dermatitis [15, 27], T‐cell lymphomas [6, 14, 29, 30], or psoriasis [16]. According to research conducted at the Poznan University of Medical Sciences, SLEB was detected in active lesions in 100% of patients with atopic dermatitis. In the case of inactive lesions, thin SLEB was found in 40% of the subjects, which was supposed to reflect the presence of subclinical inflammation and the readiness to develop typical skin lesions. SLEB was also observed in lesional skin in psoriasis patients, which was not observed in intact skin. It has not been observed in healthy people [16, 25]. However, in studies on patients with mycosis fungoides, the presence of SLEB was demonstrated in 100% of the subjects in the affected skin [10, 30]. In a study assessing the effectiveness of UVA1 phototherapy in patients with mycosis fungoides, a decrease in the average SLEB value and normalization of skin echogenicity along with an improvement in the local condition were observed [14, 29].

In a study assessing the effectiveness of treatment with various forms of phototherapy in patients with mycosis fungoides, the presence of SLEB was detected in 100% of the subjects before treatment was started. However, after treatment, SLEB decreased significantly in all patients, and in 66% it was no longer observed. The thickness of SLEB correlated with the severity of the disease [30]. In turn, in psoriasis patients, it has been shown that the thickness of SLEB decreases with a decrease in the PASI (Psoriasis Area and Severity Index), an indicator of the extent and severity of skin lesions in psoriasis [16]. HFUS may also be useful for noninvasive and objective assessment of skin lesions during radiotherapy in patients with head and neck cancer—SLEB was not observed before the initiation of treatment, but it was detected during and immediately after irradiation. Three months after the end of radiotherapy, the presence of SLEB in the irradiated skin was not detected [26].

Moreover, HFUS image showed correlation with the histological examination of lesional skin samples taken from patients with atopic dermatitis or mycosis fungoides [10, 15]. In patients with active atopic dermatitis, not only SLEB but also decreased skin echogenicity was observed. After implementing effective treatment, SLEB disappeared and echogenicity normalized [14]. Reduced echogenicity of the skin may be a consequence of not only swelling, but also of an increased number of inflammatory or cancer cells or the destruction of elastin fibers. In patients with atopic dermatitis, it was observed that the affected skin before treatment was less echogenic than after [6, 15]. In our study group, reduced echogenicity was observed in one patient with ACLE (50%) and in three patients with SCLE (23.1%), but without the presence of SLEB. It was also found in 11 patients with CCLE (26.2%), and in 4 of them (9.5%) SLEB was also present. However, no statistically significant correlation was found between CLASI‐A or CLASI‐D and decreased echogenicity.

In HFUS images, diseases involving skin sclerosis are characterized by increased echogenicity, which results from the accumulation of collagen fibers. Sometimes this symptom coexists with increased skin thickness, as for example in morphea [31]. Also in scarring alopecia, where fibrosis and atrophy of hair follicles occurs, increased echogenicity is observed [12, 32]. In the present study, increased echogenicity was found in the CCLE group, but only in DLE and lupus erythematosus panniculitis (LEP) subtypes, with some of them diagnosed as a scar, but also in the active phase. As it is known, changes typical of ACLE and SCLE do not subside with either atrophy or scarring, but it is a symptom typical for DLE [1, 3]. It should be noted that in this analysis, there was a statistically significant inverse correlation between increased echogenicity and CLASI‐A (*p* = 0.004)—greater skin echogenicity was associated with lower disease activity, which may be an expression of progressive atrophy and scarring. It can therefore be assumed that an increase in echogenicity within the active CLE lesion may indicate a gradual cessation of activity and development of scar tissue, but this is only a hypothesis that requires histological confirmation.

In our analysis, in some patients, skin thickening was observed. The average skin thickness of active lesions was significantly greater than the average thickness of healthy skin (*p* = 0.001). However, no statistically significant differences were observed in active and inactive lesions and in inactive lesions compared to healthy skin. There was also no correlation between skin thickness and CLASI‐A. On the other hand, in early SSc, lasting less than 2 years, higher skin thickness and lower skin echogenicity are observed, which may reflect the edematous phase. The degree of skin thickening tends to increase with longer duration of the disease, according to the progression toward fibrosis and ultimately atrophy [7, 8]. The role of skin thickness requires further studies in a larger population of patients, especially in the CCLE group.

The limitations of our study are a small group of patients with ACLE, and lack of standardization in performing and evaluating HFUS in CLE. Potential differences in ultrasound device, including frequency and settings may affect the interpretation of results.

To sum up, the largest deviations in HFUS were observed in patients with CCLE, less severe in patients with SCLE, and the smallest in patients with ACLE. SLEB, which in our opinion seems to be the most useful parameter, is observed only in patients with active disease. The SLEB value also shows a significant correlation with the CLASI‐A value—increasing CLASI‐A by one increases the expected SLEB value by 0.012 mm. A statistically significant inverse correlation was also demonstrated between CLASI‐A and increased skin echogenicity—as the CLASI‐A value decreases, skin echogenicity increases significantly.

## 5 Conclusions

A SLEB in the HFUS image of the skin was observed only in patients with active CLE and its thickness correlated with CLASI‐A, which means that this band can be treated as an indicator of the activity of the disease process. The presence of SLEB in CLE patients has not been described in the scientific literature so far. We plan to conduct observations on a larger group of patients in the future. It can also be assumed that the increase in echogenicity within an active CLE focus may indicate a gradual extinction of activity and scar development, but it is only a hypothesis that requires histological confirmation. It also seems important to conduct research on the usefulness of HFUS in monitoring therapeutic effects in CLE, as well as the relationship of the HFUS image with the histological image. HFUS allows the assessment of certain features of healthy and diseased skin without performing a biopsy, but it cannot replace histological examination in the case of CLE diagnosis. HFUS may be a useful diagnostic method to assess the clinical stage of the disease and to monitor its activity and treatment.

## Conflicts of Interest

The authors declare no conflicts of interest.

## Data Availability

The data that support the findings of this study are available from the corresponding author upon reasonable request.
